# The Bioactive Extract of *Pinnigorgia* sp. Induces Apoptosis of Hepatic Stellate Cells via ROS-ERK/JNK-Caspase-3 Signaling

**DOI:** 10.3390/md16010019

**Published:** 2018-01-09

**Authors:** Liang-Mou Kuo, Po-Jen Chen, Ping-Jyun Sung, Yu-Chia Chang, Chun-Ting Ho, Yi-Hsiu Wu, Tsong-Long Hwang

**Affiliations:** 1 Graduate Institute of Clinical Medical Sciences, College of Medicine, Chang Gung University, Taoyuan 333, Taiwan; kuo33410@yahoo.com.tw; 2 Department of General Surgery, Chang Gung Memorial Hospital, Chiayi 613, Taiwan; 3 Graduate Institute of Natural Products, College of Medicine, Chang Gung University, Taoyuan 333, Taiwan; litlep@hotmail.com (P.-J.C.); Tsudanyer@hotmail.com (C.-T.H.); modemtw@gmail.com (Y.-H.W.); 4 Chinese Herbal Medicine Research Team, Healthy Aging Research Center, Chang Gung University, Taoyuan 333, Taiwan; 5 Graduate Institute of Marine Biology, National Dong Hwa University, Pingtung 944, Taiwan; pjsung@nmmba.gov.tw; 6 National Museum of Marine Biology and Aquarium, Pingtung 944, Taiwan; jay0404@gmail.com; 7 Chinese Medicine Research and Development Center, China Medical University Hospital, Taichung 404, Taiwan; 8 Graduate Institute of Natural Products, Kaohsiung Medical University, Kaohsiung 807, Taiwan; 9 Department of Marine Biotechnology and Resources, National Sun Yat-sen University, Kaohsiung 804, Taiwan; 10 Doctoral Degree Program in Marine Biotechnology, National Sun Yat-sen University & Academia Sinica, Kaohsiung 804, Taiwan; 11 Research Center for Chinese Herbal Medicine, Research Center for Food and Cosmetic Safety, Graduate Institute of Health Industry Technology, College of Human Ecology, Chang Gung University of Science and Technology, Taoyuan 333, Taiwan; 12 Department of Anaesthesiology, Chang Gung Memorial Hospital, Taoyuan 333, Taiwan

**Keywords:** hepatic stellate cells, *Pinnigorgia* sp., ROS, apoptosis, caspase-3, MAPK

## Abstract

The activation of hepatic stellate cells (HSCs) is a significant phenomenon during the pathogenesis of liver disorders, including liver cirrhosis and fibrosis. Here, we identified that the extract from a gorgonian coral *Pinnigorgia* sp. (Pin) induced apoptosis of HSC-T6 cells. Pin inhibited the viability of HSC-T6 cells and increased their subG1 population, DNA fragmentation, caspase-3 activation, and reactive oxygen species (ROS) production in a concentration-dependent manner. The Pin-induced ROS generation and apoptotic effects were significantly reversed by a thiol antioxidant, *N*-acetylcysteine (NAC). Additionally, Pin induced ERK/JNK phosphorylation and pharmacological inhibition of ERK/JNK rescued the Pin-induced cell death. Pin-activated ERK/JNK were significantly reduced after the administration of NAC; however, the inhibition of ERK/JNK failed to change the Pin-induced ROS production. Similarly, pinnigorgiol A, a pure compound isolated from Pin, elicited ROS production and apoptosis in HSC-T6 cells. The pinnigorgiol A-induced apoptosis was retrained by NAC. Together, it appears that Pin leads to apoptosis in HSC-T6 cells through ROS-mediated ERK/JNK signaling and caspase-3 activation. Pinnigorgiol A serves as a bioactive compound of Pin and may exhibit therapeutic potential by clearance of HSCs.

## 1. Introduction

Hepatic fibrosis is an early significant lesion in chronic liver diseases such as liver cirrhosis and hepatocellular carcinoma. The excessive activation and proliferation of hepatic stellate cells (HSCs) gives rise to pro-inflammatory growth factors and extracellular matrix proteins during the pathogenesis of hepatic fibrosis, initiating matrix deposition in the liver [1,2,3]. Emerging evidences reveal that the restriction of activated HSCs is a potential strategy for hepatic fibrosis therapy via repressing proliferation or inducing apoptosis of HSCs [4,5,6,7]. So far, natural products derived from marine organisms display various potential pharmacological activities, including those applicable in liver disorder [8,9,10]. It is desirable to develop novel and useful marine natural products as better remedial strategies for hepatic disease through reversing the activated HSCs back to their quiescent status.

**Figure 1:**
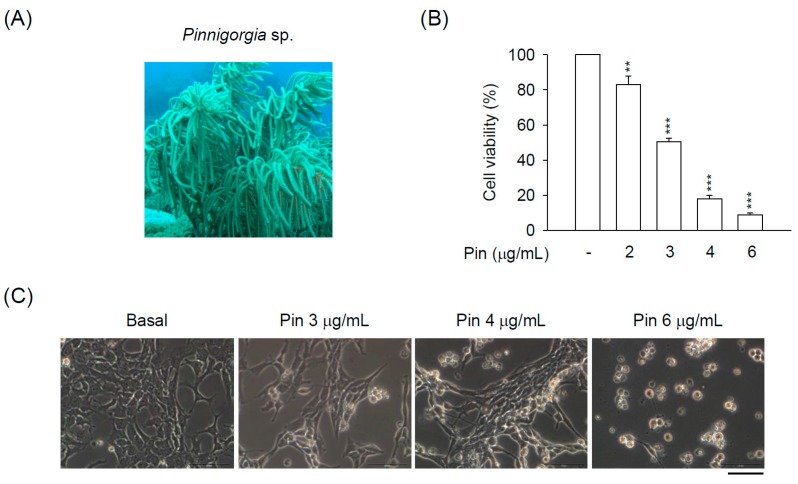

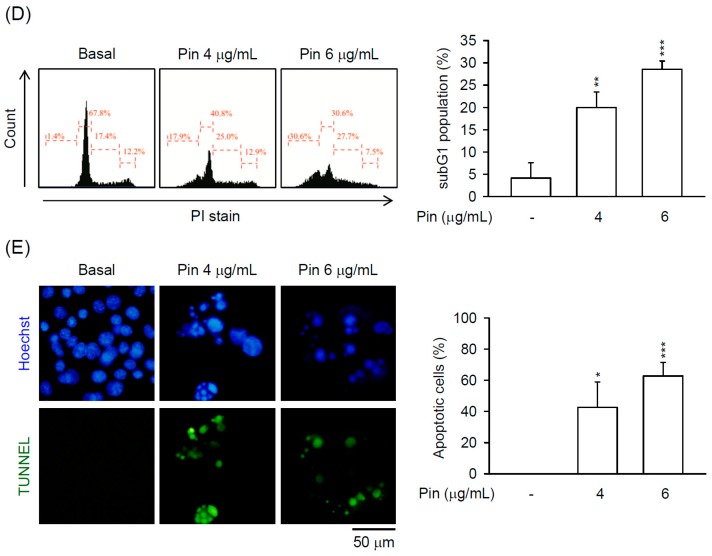
Pin elicits apoptosis in hepatic stellate cells (HSCs). HSC-T6 cells were treated with Pin (0–6 µg/mL) for 24 h. (**A**) Gorgonian coral *Pinnigorgia* sp.; (**B**) Cytotoxicity assay was monitored spectrophotometrically at 450 nm; (**C**) Cell retraction, bubbling, and apoptotic bodies were observed using microscopy; (**D**) SubG1 population was examined by propidium iodide (PI) staining and flow cytometry; (**E**) The Pin-induced apoptosis of HSC-T6 cells was determined by terminal deoxynucleotidyl transferase dUTP nick end labeling (TUNEL) assay (green). Hoechst 33,342 (blue) was used to visualize the cell nucleus. All data are expressed as the mean ± S.E.M. (*n* = 3). * *p* < 0.05, ** *p* < 0.01, *** *p* < 0.001 compared with the basal.

The *Pinnigorgia* sp. (Figure 1A) was first isolated and identified as in Taiwan [11]. Taxonomically, gorgonian coral *Pinnigorgia* sp. belongs to the animal kingdom (Kingdom Animalia), Cnidaria (Phylum Cnidaria), Anthozoa (Class Anthozoa), Octocorals subclass (Subclass Octocorallia), Gorgonian head (Order Gorgonacea), Holaxonia suborder, Family Gorgoniidae. Recently, various natural compounds from a gorgonian coral *Pinnigorgia* sp. have been shown to exhibit anti-inflammatory potential in human neutrophils [11,12,13,14,15]. In addition, the coralline extract from *Pinnigorgia* sp. (Pin) reduces the survival rate of HSCs [11,16], although the underlying mechanisms are still elusive. Therefore, it can be suggested that Pin may possess potential application to attenuate liver fibrosis via retraining the HSCs activation.

The aim of this study was to uncover the pharmacological mechanism of Pin-inhibited cell viability in a rat HSC cell line, HSC-T6 cells. During proliferation and activation of HSCs, intracellular redox homeostasis tightly controls the cell viability. Intracellular glutathione (GSH) level is an important factor to modulate redox balance [17]. GSH depletion induces reactive oxygen species (ROS) accumulation, leading to caspase-3-dependent apoptosis in HSCs [18,19,20]. Moreover, mitogen-activated protein kinases (MAPKs) signaling is also involved in redox homeostasis and apoptosis in HSCs [20,21,22,23].

In the present study, we found that the Pin-repressed cell viability in HSCs was dependent on caspase-3-, ROS-, and ERK/JNK-mediated apoptosis. Pin-induced ERK/JNK activation occurred through ROS production. The caspase-3 inhibitor, thiol antioxidants, and ERK/JNK inhibitors significantly abolished the Pin-induced cell death in HSCs. Moreover, pinnigorgiol A served as a bioactive compound of Pin that triggered the apoptosis and ROS production in HSCs. Our findings suggest that marine natural products from gorgonian coral including Pin and pinnigorgiol A exhibit therapeutic potentials by clearance of HSCs.

## 2. Results

### 2.1. Pin Exhibits Caspase-3-Dependent Apoptosis in HSC-T6 Cells

Recently, the extract from a gorgonian coral *Pinnigorgia* sp. (Pin) was isolated and found to suppress the cell viability of rat HSC-T6 cells [11]. However, the pharmacological mechanism of Pin-induced suppression of HSC-T6 cell viability is still undiscovered. Here, we studied the detailed mechanism of Pin-inhibited viability in HSCs. Pin inhibited the cell viability of HSC-T6 cells in a concentration-dependent manner (2–6 μg/mL) (Figure 1B). To monitor the Pin-induced cell death in HSC-T6 cells, we observed the changes in cellular morphology. Pin (3–6 μg/mL) gradually led to the apoptotic changes of HSC-T6 cells, including cell shrinkage and the formation of membrane blebs, apoptotic body, and cell debris (Figure 1C). To further determine the apoptotic features, flow cytometry and terminal deoxynucleotidyl transferase (TdT) dUTP nick-end labeling (TUNEL) staining were assayed. Pin (4 and 6 μg/mL) apparently increased the subG1 population, which was as an indicator of apoptosis. Also, Pin (4 and 6 μg/mL) induced DNA fragmentation in HSC-T6 cells (Figure 1D,E).

**Figure 2:**
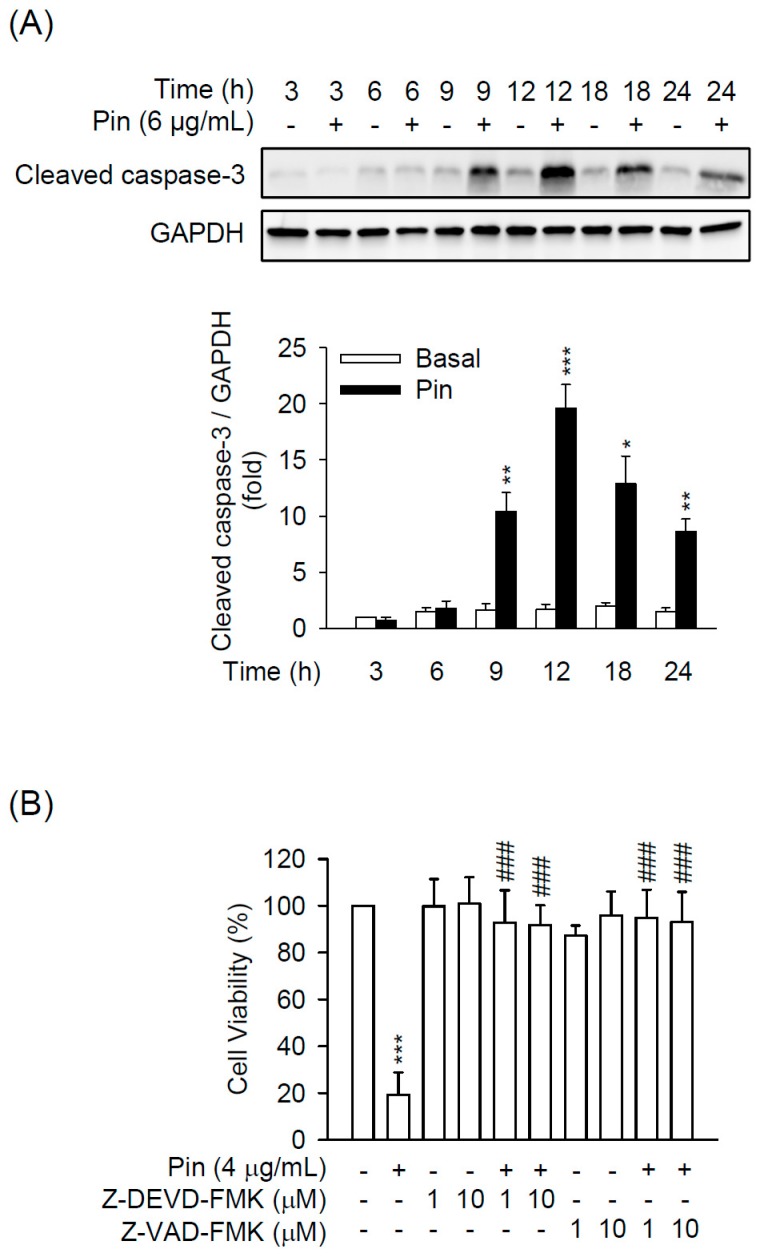
Pin-induced apoptosis is dependent on caspase-3 activation in HSCs. (**A**) HSC-T6 cells were treated with Pin (6 µg/mL) for 3–24 h. Cleaved caspase-3 and glyceraldehyde-3-phosphate dehydrogenase (GAPDH) were analyzed by immunoblot analysis using antibodies against cleaved caspase-3 or GAPDH. Quantitation of the cleaved caspase-3/GAPDH ratio is shown; (**B**) HSC-T6 cells were pretreated with Z-DEVD-FMK (caspase-3 inhibitor) or Z-VAD-FMK (general caspase inhibitor) for 1 h. Subsequently, HSC-T6 was incubated with Pin (4 µg/mL) for 24 h. The cytotoxicity assay was monitored spectrophotometrically at 450 nm. All data are expressed as the mean ± S.E.M. (*n* = 3). * *p* < 0.05, ** *p* < 0.01, *** *p* < 0.001 compared with the basal; ^###^ *p* < 0.001 compared with the Pin alone.

Caspase-3 plays a central role in apoptotic responses. As shown in the results, the expression of cleaved caspase-3 was significantly increased in Pin (6 μg/mL)-treated HSC-T6 cells during 9–24 h (Figure 2A). Moreover, the Pin-inhibited cell viability in HSC-T6 cells was blocked by pre-treatment of the general caspase inhibitor, Z-VAD-FMK, or the specific caspase-3 inhibitor, Z-DEVD-FMK (Figure 2B), suggesting that Pin-induced apoptosis in HSC-T6 cells occurs through caspase-3-dependent pathway.

### 2.2. Pin-Induced Apoptosis Is Dependent on Intracellular ROS Production in HSC-T6 Cells

**Figure 3:**
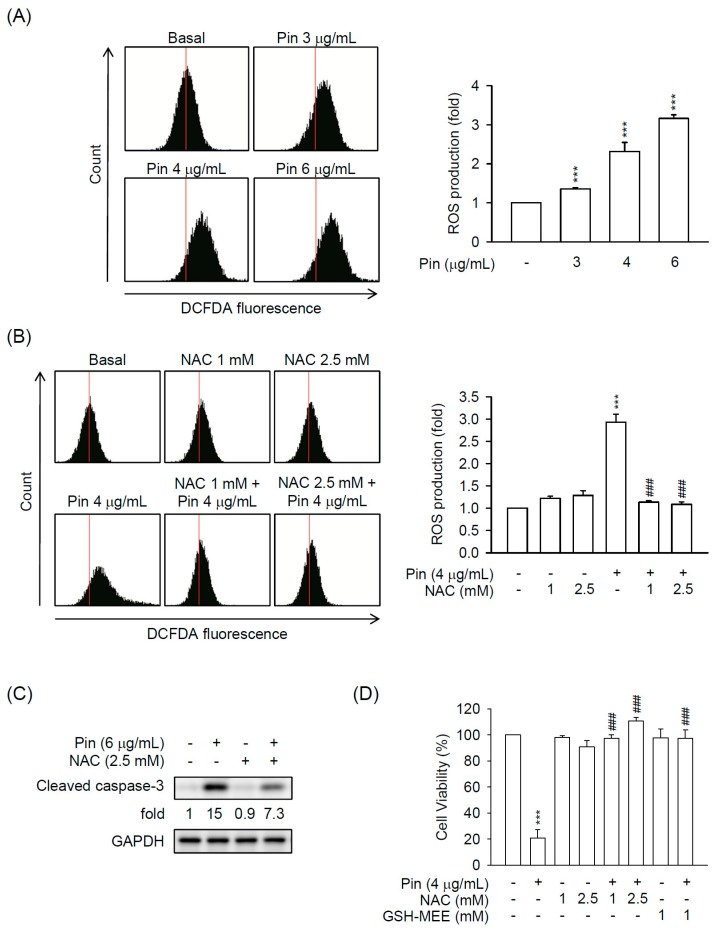
Pin-induced apoptosis relies on intracellular reactive oxygen species (ROS) production in HSCs. (**A**) HSC-T6 cells were treated with Pin (3–6 µg/mL) for 6 h. Intracellular ROS was determined by 2′,7′-dichlorofluorescein diacetate (DCFDA) assay using flow cytometry; (**B**) HSC-T6 cells were pretreated with *N*-acetylcysteine (NAC; 1 and 2.5 mM for 1 h) before the addition of Pin (4 µg/mL for 6 h). Intracellular ROS was determined by DCFDA assay using flow cytometry; (**C**) HSC-T6 cells were pretreated with NAC (2.5 mM) for 1 h and then exposed to Pin (6 µg/mL) for 12 h. Cleaved caspase-3 and GAPDH was analyzed by immunoblot analysis using antibodies against cleaved caspase-3 or GAPDH; (**D**) HSC-T6 cells were pretreated with NAC (1 and 2.5 mM) or glutathione reduced ethyl ester (GSH-MEE) (1 mM) for 1 h and then exposed to Pin (4 µg/mL) for 24 h. The cytotoxicity assay was monitored spectrophotometrically at 450 nm. All data are expressed as the mean ± S.E.M. (*n* = 3). *** *p* < 0.001 compared with the basal. ^###^ *p* < 0.001 compared with the Pin alone.

Until now, GSH depletion-induced ROS accumulation is known to cause caspase-3-dependent apoptosis in HSCs [18,19]. To investigate whether Pin causes oxidative stress in HSCs, the intracellular ROS production was determined. Pin induced ROS production in HSC-T6 cells in a dose-dependent (3–6 μg/mL) manner (Figure 3A). Furthermore, the thiol-antioxidant, NAC, inhibited the Pin-induced ROS generation in HSC-T6 cells (Figure 3B). Importantly, both NAC and a permeable membrane derivative of GSH, glutathione monoethyl ester (GSH-MEE), apparently attenuated the Pin-induced caspase-3 activation, cell death, and DNA fragmentation in HSC-T6 cells (Figure 3C,D and Figure S1), suggesting that the Pin-induced ROS production is a cause of cell death in HSC-T6 cells.

### 2.3. Pin-Induced Cell Death of HSC-T6 Cells Is through ERK and JNK Pathway

**Figure 4:**
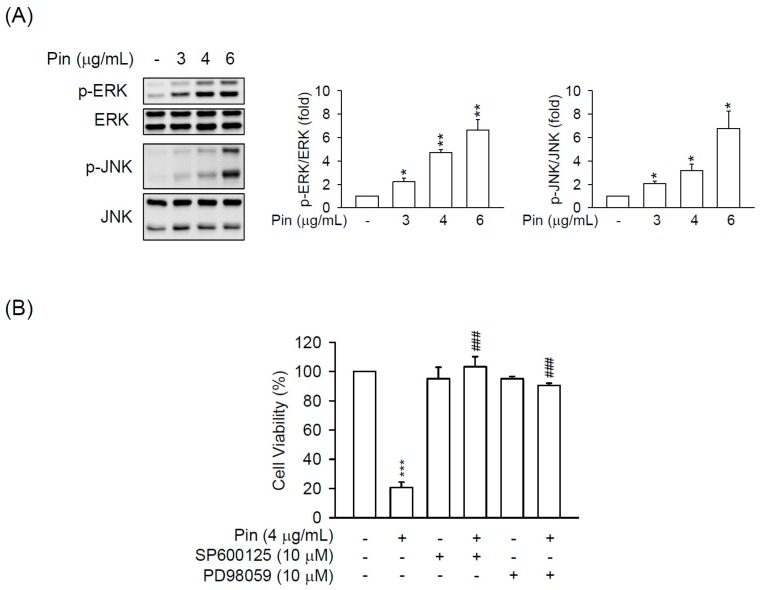
Pin-induced apoptosis is mediated through JNK and ERK pathway in HSCs. (**A**) HSC-T6 cells were treated with Pin (3–6 µg/mL) for 6 h. Phosphorylation of ERK and JNK were analyzed by immunoblot analysis using antibodies against p-JNK or p-ERK. Quantitation of the p-JNK/JNK and p-ERK/ERK ratio was shown; (**B**) HSC-T6 cells were pretreated with SP600125 (JNK inhibitor; 10 µM) or PD98059 (ERK inhibitor; 10 µM) for 1 h and then exposure to Pin (4 µg/mL) for 24 h. Cytotoxicity assay was monitored spectrophotometrically at 450 nm. All data are expressed as the mean ± S.E.M. (*n* = 3). * *p* < 0.05, ** *p* < 0.01, *** *p* < 0.001 compared with the basal. ^###^ *p* < 0.001 compared with the Pin alone.

The significance of MAPKs pathway in cell growth and apoptosis has been well established [24]. Therefore, we next examined whether MAPKs is involved in the Pin-induced apoptosis in HSC-T6 cells. Pin (6 μg/mL) significantly induced the phosphorylation of ERK and JNK but not p38 MAPK in HSC-T6 cells for 3–12 h (Figure S2A). In addition, Pin led to ERK and JNK activation in HSC-T6 cells in a dose-dependent manner. The pharmacological inhibitors of JNK (SP600125) and ERK (PD98059) completely blocked the Pin-repressed cell viability in HSC-T6 cells (Figure 4), suggesting the critical role of ERK and JNK activation in Pin-induced apoptosis in HSCs. In agreement with the inactivation of p38 MAPK in Pin-treated HSC-T6 cells, the inhibitors of p38 (SB202190 and 203580) failed to restrict the Pin-induced cell death (Figure S2B).

### 2.4. Pin-Activated ERK and JNK Pathway Act as Downstream Signals of ROS Production in HSC-T6 Cells

**Figure 5:**
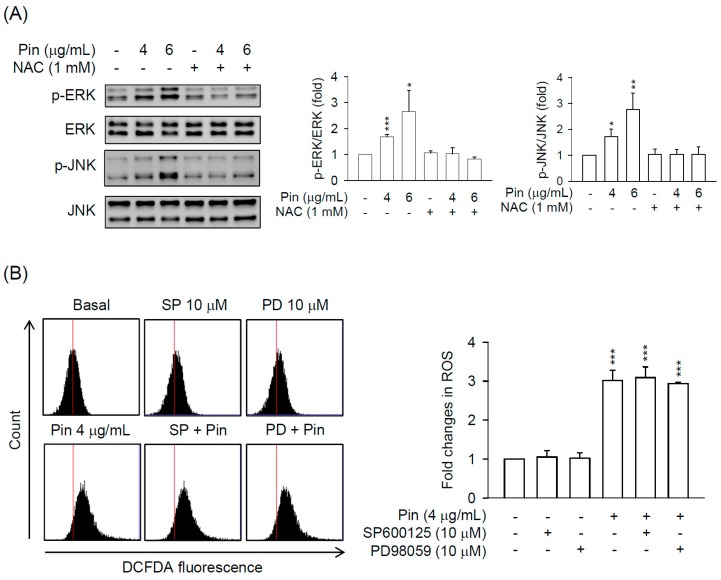
Pin-activated ERK and JNK signaling is triggered by ROS production in HSCs. (**A**) HSC-T6 cells were pretreated with or without NAC (1 mM) for 1 h and then exposed to Pin (4 or 6 µg/mL) for 6 h. Phosphorylation of ERK and JNK were analyzed by immunoblot analysis using antibodies against p-JNK or p-ERK. Quantitation of the p-JNK/JNK and p-ERK/ERK ratio was shown; (**B**) HSC-T6 cells were pretreated with SP600125 (JNK inhibitor; 10 µM) or PD98059 (ERK inhibitor; 10 µM) for 1 h and then exposed to Pin (4 µg/mL) for 6 h. Intracellular ROS was determined by DCFDA assay using flow cytometry. All data are expressed as the mean ± S.E.M. (*n* = 3). * *p* < 0.05, ** *p* < 0.01, *** *p* < 0.001 compared with the basal.

It has been documented that ERK may be an upstream regulator of ROS-induced apoptosis [25], however, different studies have reported conflicting findings [26]. To clarify the relationship between ROS and ERK/JNK in Pin-treated HSC-T6 cells, the HSC-T6 cells were pre-treated with inhibitors of ERK/JNK or thiol-antioxidant to examine it. As shown in the results, NAC evidently restrained the Pin-induced ERK and JNK activation in HSC-T6 cells (Figure 5A). In contrast, the inhibitors of JNK (SP600125) and ERK (PD98059) failed to alter Pin-induced ROS production in HSC-T6 cells (Figure 5B). Taken together, we propose that Pin stimulates the ROS production and subsequently activates ERK/JNK signaling leading to caspase-3-dependent cell death in HSC-T6 cells.

### 2.5. Pinnigorgiol a Serves as a Bioactive Component of Pin to Trigger the ROS-Dependent Apoptosis in HSC-T6 Cells

**Figure 6:**
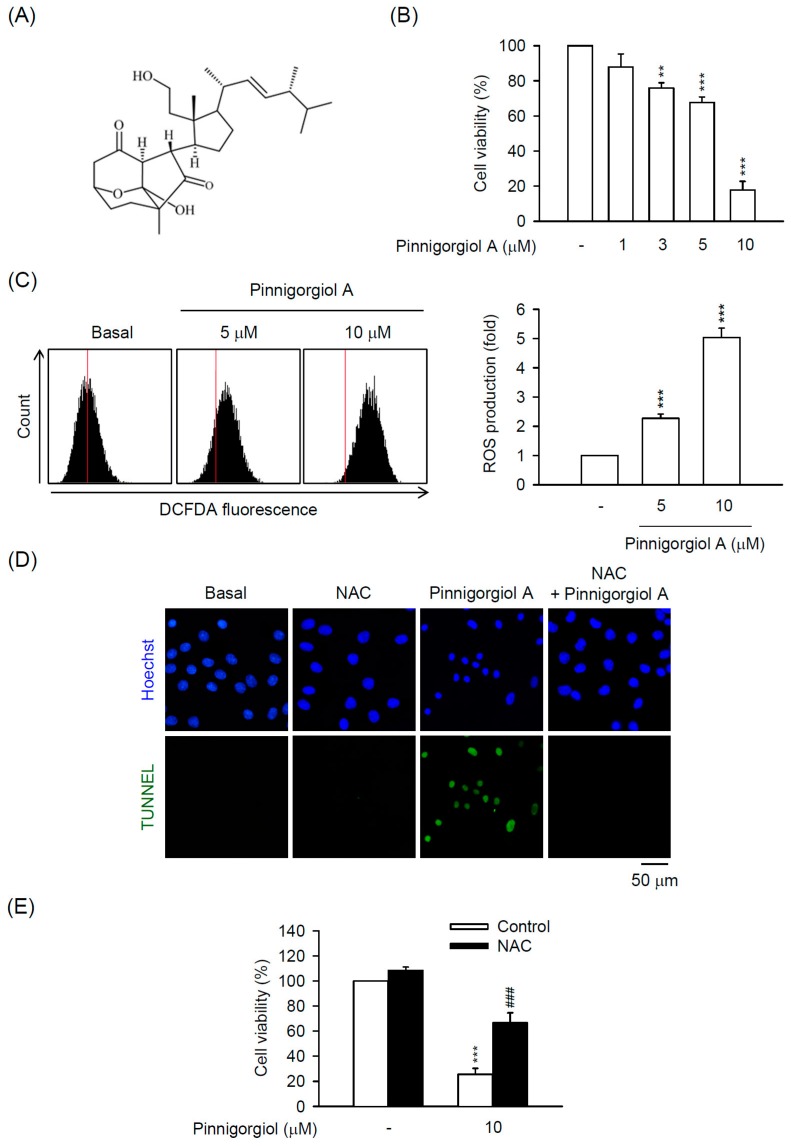
Pinnigorgiol A exhibits ROS-dependent apoptosis in HSCs. (**A**) Chemical structure of pinnigorgiol A; (**B**) HSC-T6 cells were treated with pinnigorgiol A (1–10 µM) for 24 h. Cytotoxicity assay was monitored spectrophotometrically at 450 nm; (**C**) HSC-T6 cells were treated with pinnigorgiol A (5 or 10 µM) for 6 h. Intracellular ROS was determined by DCFDA assay and flow cytometry; (**D**) HSC-T6 cells were treated with pinnigorgiol A (10 µM) for 12 h. Apoptotic effect of pinnigorgiol A was determined by TUNEL assay (green). Hoechst 33,342 (blue) was used to visualize the cell nucleus; (**E**) HSC-T6 cells were pretreated with NAC (2.5 mM) for 1 h and then incubated with pinnigorgiol A (10 µM) for 24 h. Cytotoxicity assay was monitored spectrophotometrically at 450 nm. All data are expressed as the mean ± S.E.M. (*n* = 3). ** *p* < 0.01; *** *p* < 0.001 compared with the basal. ^###^ *p* < 0.001 compared with the pinnigorgiol A alone.

Three novel 9,11-secosterols, pinnigorgiol A–C, were isolated from Pin to test the viability of HSC-T6 cells. Among them, pinnigorgiol A and B displayed inhibitory effect on the cell viability with an IC_50_ value of 5.77 ± 0.27 and 7.89 ± 0.52 µM, respectively, but pinnigorgiol C had no effect on cell viability [16]. Pinnigorgiol A (Figure 6A) exhibited the maximum inhibitory effect on the cell viability of HSC-T6 cells; however, the pharmacological mechanism is still unknown. As shown in Figure 6B, pinnigorgiol A (1–10 µM) inhibited the cell viability of HSC-T6 cells in a dose-dependent manner. Pinnigorgiol B (3–10 µM) also dose-dependently repressed the cell viability of HSC-T6 cells (Figure S3); however, the inhibitory effect was weaker than pinnigorgiol A. Further, pinnigorgiol A induced ROS production and DNA fragmentation in HSC-T6 cells. Furthermore, the pinnigorgiol A-induced apoptotic effect and cell death were significantly attenuated by the thio-antioxidant NAC in HSC-T6 cells (Figure 6C–E), suggesting that the cytotoxic effect of Pin and pinnigorgiol A is comparable in HSCs. In summary, we propose that pinnigorgiol A may be a representative bioactive component of Pin that elicits apoptosis in HSCs through ROS-ERK/JNK-caspase-3 signal pathway and may offer a possible medical application in liver pathogenesis.

## 3. Discussion

The activation of HSCs is one of the major contributors to hepatic fibrosis. Emerging evidences show that promoting cell death of HSCs is a potential strategy in the remission of liver fibrosis [4,5,27,28]. In the present study, our data revealed that Pin significantly induced cell apoptosis in HSCs in a concentration-dependent way. Pin significantly exhibited an inhibitory effect on the cell viability in HSC-T6 cells with apparent apoptotic changes, including cell shrinkage, chromatin condensation, and membrane blebs and apoptotic bodies formation (Figure 1). Moreover, it has been proven that DNA fragmentation usually accompanies increased sub-G1 population and activated caspase-3 in apoptotic cells [29,30,31]. We also found that Pin increased the sub-G1 population, DNA fragmentation, and caspase-3 activation in HSC-T6 cells (Figure 1 and Figure 2A), suggesting that the Pin-repressed HSCs viability is dependent on caspase-3-mediated apoptosis. In agreement with this proposal, the apoptotic effects of Pin were completely reversed by Z-DEVD-FMK, a specific caspase-3 inhibitor (Figure 3B). Activation of caspase-3 has a pivotal role in the apoptotic responses. Thus, Pin stimulated the apoptotic process in HSCs through caspase-3-dependent pathway. These findings suggest a possibility of Pin to restrict HSCs activation during liver pathogenesis.

Oxidative stress can induce the execution of the apoptotic pathway and excessive ROS production will lead to aggravation of apoptosis [32,33,34]. So far, ROS generation has been documented to cause apoptosis in HSCs [18,20,35]. In support of this theory, intracellular ROS was increased by treatment of Pin in HSC-T6 cells that was significantly reversed by a thiol-based antioxidant, NAC (Figure 3). In addition, GSH is another intracellular antioxidant that regulates the cell viability in HSCs and GSH displays an inhibitory effect against ROS-induced apoptotic cell death [17,18]. In addition, NAC and cell-permeable GSH (GSH-MEE) significantly blocked the Pin-inhibited cell viability in HSC-T6 cells, suggesting that the Pin-induced apoptosis is based on ROS production in HSCs.

MAPKs (ERK, JNK, and p38 MAPK) are important mediators of signal transduction under oxidative stress in HSCs to control cell growth, differentiation, survival, and apoptosis [18,36,37,38]. In our previous study, the apoptotic effect of oridonin-induced ROS in HSC-T6 cells was not affected by specific inhibitors of p38 MAPK, JNK, and ERK, respectively [20]. However, Huang and colleagues found that the oridonin-induced apoptosis and ROS production in HepG2 cells occurred through MAPK signaling pathways [37]. Thus, the role of MAPKs in the oxidative stress-elicited HSCs apoptosis is still uncertain. As the results showed, Pin induced the phosphorylation of ERK and JNK but not p38 MAPK in HSCs (Figure 4 and Figure S2). Pharmacological inhibitors of JNK (SP600125) and ERK (PD98059) rescued the Pin-induced cell apoptosis in HSCs, suggesting that the apoptotic effects of Pin in HSC were mediated by ERK and JNK. It has been documented that blocking the p38 MAPK by a specific inhibitor, SB203580, increased the proliferation in HSCs [39], whereas activation of p38 induced HSC apoptosis [10,40]. This may explain the selective inhibition of Pin on the activation of ERK and JNK but not p38 MAPK in HSCs, which may also serve as a means to explore the relationship between MAPKs and apoptosis in HSCs.

Three pure compounds, pinnigorgiol A-C, were isolated from Pin to decrease the viability of HSC-T6 cells at 10 µM. Among them, pinnigorgiol A displayed the maximum inhibitory effect on the cell viability with an IC_50_ value of 5.77 ± 0.27 µM [16]. In the present study, we unraveled that pinnigorgiol A-induced apoptosis of HSCs was also mediated by increasing ROS production (Figure 6), suggesting that pinnigorgiol A may be the representative bioactive compound of Pin leading to cell death in HSCs. To date, marine bioactive compounds have been characterized and have therapeutic potential to treat human disorders; however, the studies investigating their role in treating liver diseases are still limited [10]. Therefore, pinnigorgiol A may provide a therapeutic opportunity to retrain HSCs activation in liver fibrosis. Activation of HSCs is mediated by various mechanisms and some of them exhibit the therapeutic specificity for restricting HSC activation but not affecting hepatocytes [2]. In this study, the proof of whether Pin or pinnigorgiol A leads to apoptosis in hepatocytes is not carried out yet. The possible liver injuries by Pin or pinnigorgiol A still should be carefully dissected in the future.

In summary, our findings reveal an important example of how a novel coral extract from *Pinnigorgia* sp. (Pin) leads to a caspase-3-dependent apoptosis in HSCs via inducing ROS-mediated ERK/JNK signaling. Pinnigorgiol A may be a bioactive compound of Pin that possibly attenuates HSCs activation in liver fibrosis.

## 4. Materials and Methods

### 4.1. Reagents

Glutathione monoethyl ester (GSH-MEE) was purchased from Calbiochem (La Jolla, CA, USA). Z-DEVD-FMK and Z-VAD-FMK were obtained from BioVision (Mountain, PA, USA). The cell proliferation reagent WST-1 and RNase A were obtained from Roche Applied Sciences (Mannheim, Germany). Antibodies against cleaved caspase-3, phospho-ERK1/2, ERK1/2, phospho-JNK, and JNK were purchased from Cell Signaling (Beverly, MA, USA). Antibodies against phospho-p38 and p38 MAPK were obtained from Santa Cruz Biotechnology (Santa Cruz, CA, USA). The other chemicals were purchased from Sigma (St. Louis, MO, USA). The extraction and isolation of crude extract from *Pinnigorgia* sp. (Pin) and pinnigorgiol A were carried out as described previously [11,16]. The ^1^H and ^13^C NMR spectrum of pinnigorgiol A–C have been identified previously [16]. When Pin and pinnigorgiol A were dissolved in dimethyl sulfoxide (DMSO) (Sigma-Aldrich), the final concentration of DMSO in the cell experiments was 0.1% and did not affect the parameters measured.

### 4.2. Cell Culture

HSC-T6, a rat HSC cell line, was kindly provided by Professor Scott L. Friedman (Mount Sinai School of Medicine, NY, USA). HSC-T6 cells were cultured at 37 °C in Dulbecco’s minimum essential medium (DMEM; Gibco, Grand Island, NY, USA) supplemented with 10% fetal bovine serum (FBS) and antibiotics (100 U/mL penicillin, 100 μg/mL streptomycin, and 2.5 μg/mL amphotericin B) in a humidified atmosphere with 5% CO_2_.

### 4.3. Cell Viability Assay

Cell viability was measured using WST-1 assay. HSC-T6 cells were cultured in DMEM containing 0.5% FBS for 24 h. After incubation, cells were incubated with indicated concentrations of Pin or pinnigorgiol A for 24 h. Subsequently, the WST-1 reagent was added and incubated at 37 °C for 2 h. The absorbance was measured spectrophotometrically at 450 nm (Thermo Labsystems, Waltham, MA, USA).

### 4.4. Analysis of SubG1 Population

HSC-T6 cells were treated with Pin for 24 h and then the cells were harvested and fixed in 70% ethanol for 1 h. After that, the cells were washed with PBS twice and incubated in propidium iodide staining buffer (50 μg/mL propidium iodide, 0.1 mg/mL DNase-free RNase A, and 0.5% Triton X-100) for 30 min at 37 °C. The subG1 population was determined by measuring DNA content using flow cytometry.

### 4.5. Measurement of Intracellular ROS Generation

The intracellular ROS levels were examined using 2′,7′-dichlorofluorescein diacetate (DCF-DA; Sigma) reagent and analyzed by flow cytometry (BD Biosciences, San Jose, CA, USA).

### 4.6. Western Blotting

Cell lysates were suspended in lysis buffer (50 mM HEPES, 100 mM NaCl, 1 mM EDTA, 2 mM Na_3_VO_4_, 5% 2-mercaptoethanol, and 1% Triton-X-100), and then centrifuged at 14,000× *g* for 20 min at 4 °C. Proteins were separated by sodium dodecyl sulfate-polyacrylamide gel electrophoresis (SDS-PAGE) and electrophoresed onto a nitrocellulose membrane. Indicated primary antibodies and horseradish peroxidase-conjugated secondary antibodies were used to determine the protein levels using an enhanced chemiluminescence system. Signals were detected and quantified using a densitometer (UVP, Upland, CA, USA).

### 4.7. TUNEL Assay

The DNA fragmentation in apoptosis was detected using terminal deoxyribonucleotidyl transferase-mediated dUTP-digoxigenin nick end labeling (TUNEL) apoptosis detection kit (Roche, Mannheim, Germany). The nuclei were counterstained with DAPI. Visual images were analyzed using an OLYMPUS IX 81 microscope (Olympus, Tokyo, Japan).

### 4.8. Statistical Analysis

Data were expressed as the mean ± standard error of mean (SEM). Statistical comparisons were made between two groups using Student’s *t*-test. A value of *p* < 0.05 was considered statistically significant.

## 5. Conclusions

Clearance of activated HSCs is a potential strategy to treat liver fibrosis by pharmacologically prompting apoptosis. In the present study, we found that the extract from a gorgonian coral *Pinnigorgia* sp. (Pin) led to caspase-3-dependent apoptosis in HSCs that is modulated via ROS production and subsequent ERK/JNK activation. Moreover, the Pin-derived pure compound, pinnigorgiol A, was identified as a bioactive component which elicited apoptosis and ROS production in HSCs. Altogether, we uncovered the molecular mechanisms of the marine natural products, Pin and pinnigorgiol A, and predicted their therapeutic potential eliminating HSCs in liver diseases.

## Supplementary Materials

The following are available online at www.mdpi.com/1660-3397/16/1/19/s1, Figure S1. *N*-acetylcysteine (NAC) abolishes the Pin-induced apoptosis in hepatic stellate cells (HSCs), Figure S2. Pin-induced apoptosis is independent of p38 MAPK in HSCs, Figure S3. Pinnigorgiol B inhibits the cell viability of HSCs.

## References

[1] Yin C., Evason K.J., Asahina K., Stainier D.Y. (2013). Hepatic stellate cells in liver development, regeneration, and cancer. J. Clin. Investig..

[2] Tsuchida T., Friedman S.L. (2017). Mechanisms of hepatic stellate cell activation. Nat. Rev. Gastroenterol. Hepatol..

[3] Tacke F., Trautwein C. (2015). Mechanisms of liver fibrosis resolution. J. Hepatol..

[4] Friedman S.L. (2010). Evolving challenges in hepatic fibrosis. Nat. Rev. Gastroenterol. Hepatol..

[5] Huang Y., Deng X., Liang J. (2017). Modulation of hepatic stellate cells and reversibility of hepatic fibrosis. Exp. Cell Res..

[6] Zhang C.Y., Yuan W.G., He P., Lei J.H., Wang C.X. (2016). Liver fibrosis and hepatic stellate cells: Etiology, pathological hallmarks and therapeutic targets. World J. Gastroenterol..

[7] Fallowfield J.A. (2011). Therapeutic targets in liver fibrosis. Am. J. Physiol. Gastrointest. Liver Physiol..

[8] Molinski T.F., Dalisay D.S., Lievens S.L., Saludes J.P. (2009). Drug development from marine natural products. Nat. Rev. Drug Discov..

[9] Fitton J.H. (2011). Therapies from fucoidan; multifunctional marine polymers. Mar. Drugs.

[10] Nair D.G., Weiskirchen R., Al-Musharafi S.K. (2015). The use of marine-derived bioactive compounds as potential hepatoprotective agents. Acta Pharmacol. Sin..

[11] Chang Y.C., Kuo L.M., Hwang T.L., Yeh J., Wen Z.H., Fang L.S., Wu Y.C., Lin C.S., Sheu J.H., Sung P.J. (2016). Pinnisterols A–C, New 9,11-Secosterols from a Gorgonian *Pinnigorgia* sp.. Mar. Drugs.

[12] Chang H.H., Chang Y.C., Chen W.F., Hwang T.L., Fang L.S., Wen Z.H., Chen Y.H., Wu Y.C., Sung P.J. (2016). Pubinernoid A and Apo-9′-fucoxanthinone, Secondary Metabolites from a Gorgonian Coral *Pinnigorgia* sp.. Nat. Prod. Commun..

[13] Chang Y.C., Hwang T.L., Sheu J.H., Wu Y.C., Sung P.J. (2016). New Anti-Inflammatory 9,11-Secosterols with a Rare Tricyclo [5,2,1,1] decane Ring from a Formosan Gorgonian *Pinnigorgia* sp.. Mar. Drugs.

[14] Chang Y.C., Hwang T.L., Chao C.H., Sung P.J. (2017). New Marine Sterols from a Gorgonian *Pinnigorgia* sp.. Molecules.

[15] Chang Y.C., Hwang T.L., Kuo L.M., Sung P.J. (2017). Pinnisterols D–J, New 11-Acetoxy-9,11-secosterols with a 1,4-Quinone Moiety from Formosan Gorgonian Coral *Pinnigorgia* sp. (Gorgoniidae). Mar. Drugs.

[16] Chang Y., Kuo L., Su J., Hwang T., Kuo Y., Lin C., Wu Y., Sheu J., Sung P. (2016). Pinnigorgiols A–C, 9,11-secosterols with a rare ring arrangement from a gorgonian coral *Pinnigorgia* sp.. Tetrahedron.

[17] Brunati A.M., Pagano M.A., Bindoli A., Rigobello M.P. (2010). Thiol redox systems and protein kinases in hepatic stellate cell regulatory processes. Free Radic. Res..

[18] Dunning S., Ur Rehman A., Tiebosch M.H., Hannivoort R.A., Haijer F.W., Woudenberg J., van den Heuvel F.A., Buist-Homan M., Faber K.N., Moshage H. (2013). Glutathione and antioxidant enzymes serve complementary roles in protecting activated hepatic stellate cells against hydrogen peroxide-induced cell death. Biochim. Biophys. Acta.

[19] Circu M.L., Aw T.Y. (2008). Glutathione and apoptosis. Free Radic. Res..

[20] Kuo L.M., Kuo C.Y., Lin C.Y., Hung M.F., Shen J.J., Hwang T.L. (2014). Intracellular glutathione depletion by oridonin leads to apoptosis in hepatic stellate cells. Molecules.

[21] Dong H., Guo H., Liang Y., Wang X., Niu Y. (2017). Astragaloside IV synergizes with ferulic acid to suppress hepatic stellate cells activation in vitro. Free Radic. Res..

[22] Huang Y., Li X., Wang Y., Wang H., Huang C., Li J. (2014). Endoplasmic reticulum stress-induced hepatic stellate cell apoptosis through calcium-mediated JNK/P38 MAPK and Calpain/Caspase-12 pathways. Mol. Cell. Biochem..

[23] Brenner C., Galluzzi L., Kepp O., Kroemer G. (2013). Decoding cell death signals in liver inflammation. J. Hepatol..

[24] Takeda K., Naguro I., Nishitoh H., Matsuzawa A., Ichijo H. (2011). Apoptosis signaling kinases: From stress response to health outcomes. Antioxid. Redox Signal..

[25] Sun X., Zhang X., Hu H., Lu Y., Chen J., Yasuda K., Wang H. (2009). Berberine inhibits hepatic stellate cell proliferation and prevents experimental liver fibrosis. Biol. Pharm. Bull..

[26] Wasmuth H.E., Tacke F., Trautwein C. (2010). Chemokines in liver inflammation and fibrosis. Semin. Liver Dis..

[27] Puche J.E., Saiman Y., Friedman S.L. (2013). Hepatic stellate cells and liver fibrosis. Compr. Physiol..

[28] Ray K. (2014). Liver: Hepatic stellate cells hold the key to liver fibrosis. Nat. Rev. Gastroenterol. Hepatol..

[29] Kim S.Y., Kang K.L., Lee J.C., Heo J.S. (2012). Nicotinic acetylcholine receptor alpha7 and beta4 subunits contribute nicotine-induced apoptosis in periodontal ligament stem cells. Mol. Cells.

[30] Pamenter M.E., Perkins G.A., Gu X.Q., Ellisman M.H., Haddad G.G. (2013). DIDS (4,4-diisothiocyanatostilbenedisulphonic acid) induces apoptotic cell death in a hippocampal neuronal cell line and is not neuroprotective against ischemic stress. PLoS ONE.

[31] Posadas I., Santos P., Cena V. (2012). Acetaminophen induces human neuroblastoma cell death through NFKB activation. PLoS ONE.

[32] Ricci C., Pastukh V., Leonard J., Turrens J., Wilson G., Schaffer D., Schaffer S.W. (2008). Mitochondrial DNA damage triggers mitochondrial-superoxide generation and apoptosis. Am. J. Physiol. Cell Physiol..

[33] Crosas-Molist E., Fabregat I. (2015). Role of NADPH oxidases in the redox biology of liver fibrosis. Redox Biol..

[34] Wang K. (2015). Autophagy and apoptosis in liver injury. Cell Cycle.

[35] Siegmund S.V., Qian T., de Minicis S., Harvey-White J., Kunos G., Vinod K.Y., Hungund B., Schwabe R.F. (2007). The endocannabinoid 2-arachidonoyl glycerol induces death of hepatic stellate cells via mitochondrial reactive oxygen species. FASEB J..

[36] Chowdhury A.A., Chaudhuri J., Biswas N., Manna A., Chatterjee S., Mahato S.K., Chaudhuri U., Jaisankar P., Bandyopadhyay S. (2013). Synergistic apoptosis of CML cells by buthionine sulfoximine and hydroxychavicol correlates with activation of AIF and GSH-ROS-JNK-ERK-iNOS pathway. PLoS ONE.

[37] Huang J., Wu L., Tashiro S., Onodera S., Ikejima T. (2008). Reactive oxygen species mediate oridonin-induced HepG2 apoptosis through p53, MAPK, and mitochondrial signaling pathways. J. Pharmacol. Sci..

[38] Szuster-Ciesielska A., Mizerska-Dudka M., Daniluk J., Kandefer-Szerszen M. (2013). Butein inhibits ethanol-induced activation of liver stellate cells through TGF-beta, NFkappaB, p38, and JNK signaling pathways and inhibition of oxidative stress. J. Gastroenterol..

[39] Schnabl B., Bradham C.A., Bennett B.L., Manning A.M., Stefanovic B., Brenner D.A. (2001). TAK1/JNK and p38 have opposite effects on rat hepatic stellate cells. Hepatology.

[40] Jameel N.M., Thirunavukkarasu C., Wu T., Watkins S.C., Friedman S.L., Gandhi C.R. (2009). p38-MAPK- and caspase-3-mediated superoxide-induced apoptosis of rat hepatic stellate cells: Reversal by retinoic acid. J. Cell. Physiol..

