# Supplementary material for: The Bioactive Extract of Pinnigorgia sp. Induces Apoptosis of Hepatic Stellate Cells via ROS-ERK/JNK-Caspase-3 Signaling
Source: Mar Drugs. 2018 Jan 9;16(1):19. doi: 10.3390/md16010019 (PMC5793067; doi:10.3390/md16010019)
Supplement: Supplementary file 1 [file marinedrugs-16-00019-s001.pdf]

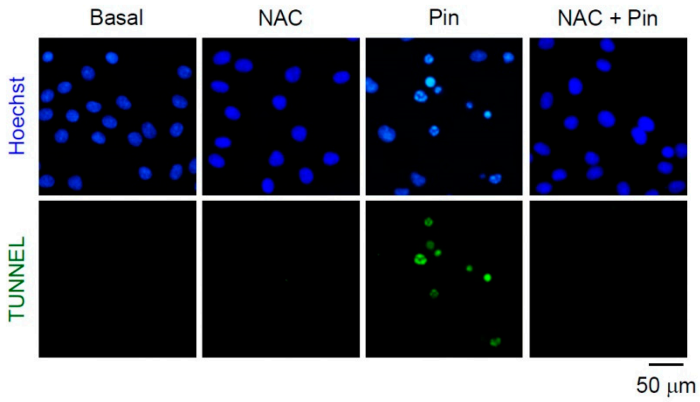

Figure S1. *N*-acetylcysteine (NAC) abolishes the Pin-induced apoptosis in hepatic stellate cells (HSCs). HSC-T6 cells were pretreated with NAC (2.5 mM) for 1 h and then incubated with Pin (6 μg/mL) for 12 h. Apoptotic effect of pinnigorgiol A were determined by terminal deoxynucleotidyl transferase dUTP nick end labeling (TUNEL) assay (green). Hoechst 33,342 (blue) was used to visualize the cell nucleus.

(A)

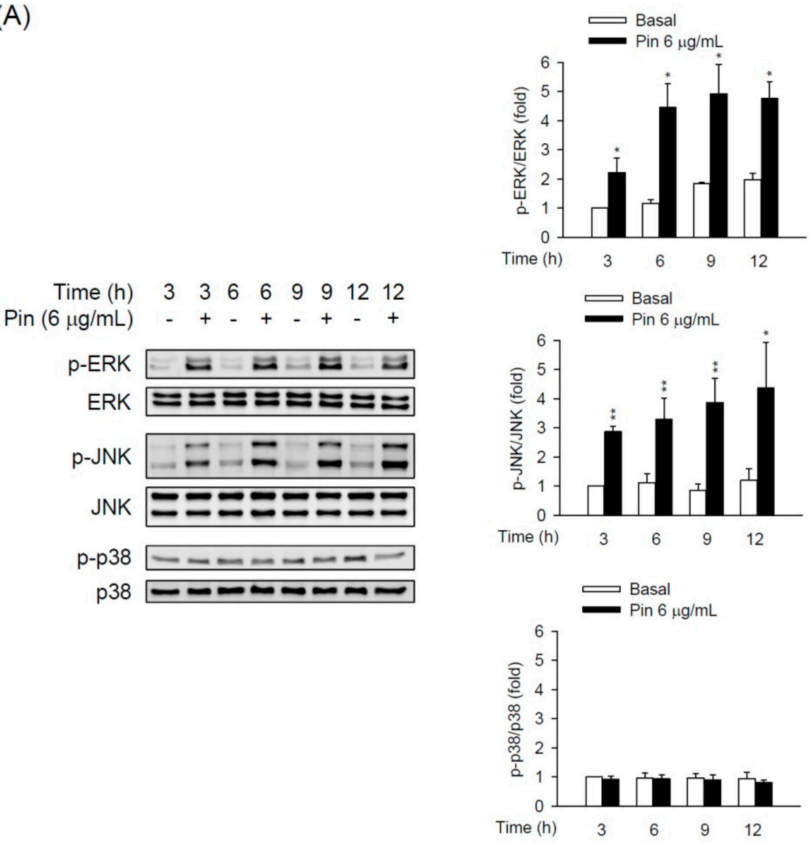

(B)

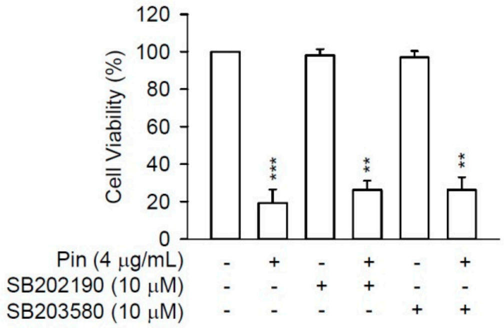

Figure S2. Pin-induced apoptosis is independent of p38 MAPK in HSCs. (**A**) HSC-T6 cells were treated with Pin (6 μg/mL) for 3–12 h. Phosphorylation of ERK, JNK and p38 were analyzed by immunoblot analysis using antibodies against p-p38, p-JNK, or p-ERK. Quantitation of the p-p38/p38, p-JNK/JNK, and p-ERK/ERK ratio was shown; (**B**) HSC-T6 cells were pretreated with SB202190 or SB203580 (p38 inhibitor; 10 μM) for 1 h and then exposed to Pin (4 μg/mL) for 24 h. Cytotoxicity assay was monitored spectrophotometrically at 450 nm. All data are expressed as the mean ± S.E.M. (n = 3). * $p < 0.05$, ** $p < 0.01$, *** $p < 0.001$ compared with the basal.

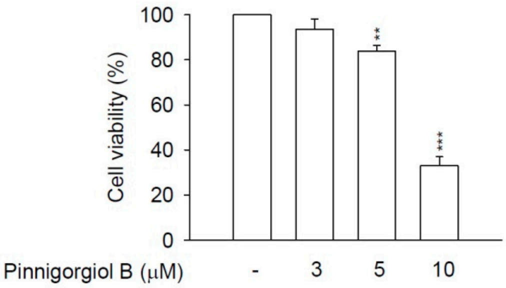

Figure S3. Pinnigorgiol B inhibits the cell viability of HSCs. HSC-T6 cells were treated with pinnigorgiol B (3–10 μM) for 24 h. Cytotoxicity assay was monitored spectrophotometrically at 450 nm.
